# Factors Influencing Italian Consumers’ Willingness to Pay for Eggs Enriched with Omega-3-Fatty Acids

**DOI:** 10.3390/foods11040545

**Published:** 2022-02-14

**Authors:** Nadia Palmieri, Walter Stefanoni, Francesco Latterini, Luigi Pari

**Affiliations:** CREA Research Centre for Engineering and Agro-Food Processing, Via della Pascolare, 16, Monterotondo, 00015 Rome, Italy; walter.stefanoni@crea.gov.it (W.S.); francesco.latterini@crea.gov.it (F.L.); luigi.pari@crea.gov.it (L.P.)

**Keywords:** consumers’ attitude, functional eggs, camelina, omega-3-fatty acids, egg quality characteristics, willingness to pay (WTP), Italy

## Abstract

This paper focused on eggs enriched with omega-3-fatty acids with the aim of understanding if functional eggs were of interest to Italian consumers, and analyzing which characteristics of table egg quality, consumers’ attitudes and socio-demographic characteristics affect the consumers’ willingness to pay (WTP) a premium price for eggs enriched with omega-3-fatty acids. We performed an online survey on 312 Italian consumers. The analysis was based on the Tobit regression model. The findings showed that unmarried females were more willing to pay a premium price for functional eggs than male consumers. Furthermore, the probability of showing a higher WTP for functional eggs increased among consumers reporting a higher income. Moreover, the WTP for functional eggs increased with the growing importance that people attributed to items such as the size of eggs, rearing type, feed given to chickens, and the provenience and brand of eggs. These results suggested that consumers need to have clear information about functional eggs. As expected, WTP for functional eggs decreased with increasing neophobia and food techno-neophobia factors. In conclusion, the findings showed an interesting potential for eggs enriched with omega-3-fatty acids, which seems to be a product with high possibility to be greatly appreciated on the market, especially if accompanied by a good, informative campaign for increasing people’s knowledge level.

## 1. Introduction

Functional foods (FFs) show the presence of technologically-developed ingredients with specific benefits for health [1], although they maintain the aspect of traditional foods [2,3].

However, functional foods are a type of novel food that do not have a long history of consumption [4]. The development and selling of functional foods is rather intricate, expensive and risky [3]. In fact, apart from potential technological obstacles, legislative issues and consumers’ demands need to be considered in the development of functional foods [3]. In particular, consumer acceptance is acknowledged as an important parameter to explore new market chances [3,5,6]. According to the current literature [7,8,9,10,11], consumer acceptance towards functional foods is mostly driven by the perceived relation between different factors, for instance diet and health [11,12]. Considering these reasons, over the last decade, an important growth of the market of functional food has been experienced in Europe [13,14], and the main markets are the UK, Germany, France and Italy [8]. Despite the development of a functional foods’ market and the economic opportunities for firms, functional foods have not yet been defined by European legislation, although there is broad consensus regarding the need for developing specific regulations, both to protect consumers and to encourage product innovation in the food industry [12]. Indeed, European legislation does not consider functional foods as a specific food category, as has happened instead for dietetic food, genetically modified organisms [15] or novel foods [16], but rather it is a concept for which there is no globally accepted definition [17,18,19].

In this framework, eggs and their enrichment via several essential nutrients are considered a suitable functional food for human consumption [20]. Enriched eggs are indeed a feasible source of omega 3 fatty acids, vitamins, several minerals, such as selenium, proteins and other different important nutrients [20,21,22,23]. According to Rokka et al. [24] hen eggs can be beneficially modified by camelina (*Camelina sativa* (L.) Crantz) seed oil, which is rich in essential omega-3-fatty acids, obtaining functional eggs that have positive effects on human health. Camelina is an oilseed belonging to the *Brassicaceae* family [25]. During the last years there has growing interest in its cultivation, mostly thanks to the short growing cycle, which allows for double cropping with widely grown species such as small grain cereal, soybean and sunflower [26]. Furthermore, camelina oil presents a particular pattern of fatty acids, which allows for different applications of this oil, i.e., animal feeding, aquaculture and veterinary [27]. On the one hand, a feature of camelina oil is its high share of omega-3 fatty acids [28]. On the other hand, it is known that the fatty acid composition of the hen egg yolk can be amended through modifications in the nutrition of the animals [24]. In fact, feeding hens with foods that are rich in long-chain omega-3-fatty acids, such as camelina, increases the amount of this kind of fatty acids within the yolk [24]. Eggs can, as a consequence, represent an alternative to fish and oilseeds, both as a source of omega-3-fatty acids [24] and also from an economic point of view due to the low price of table eggs [29]. Moreover, the introduction of egg variants (such as eggs enriched with omega-3-fatty acids) on the market should represent an interesting alternative due to the growing consumer demand for healthy and safe food [30].

Notwithstanding the interesting features of omega-3-enriched eggs mentioned in the studies above, low attention has been paid to this topic by researchers in the sector, and the consumer acceptance of eggs enriched with omega-3-fatty acids in Italy is largely unexplored. In fact, at a European level, one study [30] has focused on the preferences of Spanish consumers for these alternative types of egg, while no study has focused on Italian consumers’ preferences for functional eggs. For these reasons, and because in 2021 Italy was the fourth largest European producer of table eggs [29], this paper tries to fill a gap in the literature, with the purpose of understanding if eggs enriched with omega-3-fatty acids are of interest to Italian consumers, and analyzing which characteristics of table egg quality, consumers’ attitudes and socio-demographic characteristics affect the consumers’ willingness to pay (WTP) a premium price for eggs enriched with omega-3-fatty acids.

The paper is structured as follows. Section 2 provides a brief background on functional food. Section 3 describes the materials and methods applied. The results are presented in Section 4 and are discussed in Section 5. In addition, Section 6 concludes with some considerations.

## 2. Research Background: Consumer Motivations and Expectations towards FFs

The global growth of the functional food market is strongly related to the increased consumers’ awareness about the role of diet in maintaining human health and for the prevention of some diseases [31]. The great success of these products in terms of sales led food companies to develop foods “enriched” with beneficial substances and bioactive elements, such as antioxidants, fiber, vitamins, pro-vitamins and minerals [32].

However, despite the considerable varieties of functional foods offered in the world market, not all of them survive because they do not focus on consumers’ expectations [33].

The messages concerning functional product claims may lead to different consumer impressions [10]. Various scientific publications have shown that health is an important motivation for functional food consumption (see e.g., [2,3,34]). Indeed, people are aware that functional foods may prevent health problems and allow for a higher human wellbeing [3]. Goetzke et al. [34] found that health is a very important factor for consumers of functional food; however, their understanding of health is seen only as “small adjustments” to human health [34]. According to other authors [2], instead, the most influencing factors concerning the purchasing decisions of consumers were messages such as “functional foods are necessary” and “functional foods are a part of healthy diet”. In other words, the longing for health as well as a longer life are very effective factors, which drive consumers’ acceptance [35]. Moreover, the image of a healthy lifestyle is an important factor for consumers of functional foods [36]. In fact, according to Barauskaite et al. [36], people concerned about their image in others’ eyes buy functional food for “good impressions” of their healthy lifestyle. Moreover, Gautam et al. [37] found that beliefs about the link between nutrition and health, consumption patterns and positive attitudes towards functional food influence the consumers’ willingness to buy them.

As well as the effects on health, sensory attributes (such as taste and pleasure) and convenience of use are very important aspects for consumers of functional foods [35,38]. In fact, according to Kolbina et al. [39], people focus on both the taste and healthy aspects of functional food as the main criteria for their purchase decision. Similar results were reached by Michell’s et al. [40] study where consumers gave particular importance to the taste of functional food. Furthermore, in the Williams’ et al. [41] study, taste and smell were perceived as attributes providing additional benefits for people, while, according to Çakiroglu and Uçar [2], the taste and pleasure of a functional product as well as people awareness were among the most important factors affecting consumers’ decisions.

Moreover, packaging is an important factor for consumers of functional food [35]. In fact, according to Gutkowska and Czarnecki [42], consumers pay attention to the packaging (aesthetics as well as information placement) in the food purchase decision-making process. In addition, other marketing instruments, such as labels providing information on the potential health benefits of functional products, should affect the purchase decisions of consumers [43]. Similar results were also reached by Palmieri et al. [6], who showed that more information about functional foods should improve consumers’ willingness to pay for these new products.

Other factors that could improve consumers’ acceptance towards functional food could be “customer loyalty to a brand” and price [4]. In fact, according to some authors [44,45,46], brand can strongly influence consumers’ functional food choices. Often, consumers are more likely to accept functional foods if they are familiar with the brand selling the product [44,46]. On the other hand, the price of functional foods may also have some influence on consumer acceptance [44,46,47]. In fact, Palmieri et al. [5] showed that the potential preference for functional foods might be driven by customer loyalty to a brand and the price of the product.

Additionally, food neophobia and food techno-neophobia factors are important factors for consumers’ acceptance towards functional food [10,48]. In fact, according to Saher et al. [18], people who bought functional products were considered innovative people, even if they showed skepticism about the enrichment of food with various and new ingredients. Moreover, some consumers could be suspicious about the “unnaturalness” of functional food and, thus, perceive a possible risk associated with consumption of these products [10]. Obviously, consumers who are convinced of the safety of functional foods are more willing to consume them [49].

Finally, the acceptance of functional foods by consumers is driven by socio-demographic aspects [50]. According to some authors [4], the main drivers are age, gender, educational level, marital status and household characteristics (e.g., income and household size). In particular, according to a review study [4] in the current literature, there is not unanimous consent about a specific range age that are potential functional food consumers [4]. In fact, some studies (e.g., [47,51,52]) identified older people as the primary consumers of functional foods, while other studies (e.g., [2,53]) showed that younger people were more interested in functional foods than older people. Moreover, gender is a significant factor that influences consumers’ acceptance towards functional foods [4]. In fact, most studies [2,51,54] showed that female consumers were more likely to consume functional foods than males. Additionally, the educational level of people is an important factor in the consumers’ acceptance [4]. In fact, according to some authors [4,31], educated people showed a greater intention to purchase functional foods. Moreover, the marital status of people could affect consumers’ acceptance of functional foods [4]. In fact, according to Bekoglu et al. [48], single consumers were more likely to consume functional foods than married people, contrary to Moro et al. [55] who showed that married or widowed consumers were more willing to pay for functional foods than single or divorced people. In addition, the income of people is an important driver that influences functional food acceptance [4]. In fact, according to some authors [4,31,53,56,57] a higher income level is positively associated with higher purchase intentions of people.

## 3. Materials and Methods

### 3.1. Data Collection and the Survey

In order to identify which characteristics of table egg quality, consumers’ attitudes and socio-demographic characteristics affect the consumers’ willingness to pay (WTP) a premium price for eggs enriched with omega-3-fatty acids, a survey was carried out on a sample of 312 Italian consumers of eggs. Data were obtained through an online survey between September and November 2021. In order to reach the highest possible number of respondents, authors applied the recruitment technique of snowball sampling [58], which is based on interpersonal relations among people in the environment of social networks (i.e., Facebook, Twitter, WhatsApp and Telegram). To be involved in the survey the respondents had to fulfill the following requirements: be a consumer of eggs, be responsible for the purchase of food within his/her family and be older than 18 years old. It is important to underline that the respondents were not compensated for their participation in the survey.

The questionnaire was organized into three parts, which were: (1) consumers’ habits, their preferences and their attitudes about food choice; (2) consumers’ perceptions and their inclination regarding functional eggs; (3) socioeconomic information of respondents. In addition, the first two sections were structured on a ten-point evaluation scale (from 1 for the least liked to 10 for the most liked).

Within the first part of the questionnaire, consumers’ food habits (in terms of frequency of some food consumption), their lifestyles and their health state were investigated [4]. Moreover, following some studies focused on consumers’ behavior (see e.g., [4,59]), the importance for consumers of the health features of food, its provenience and tradition, as well as the environmental implications of food choices were analyzed. Moreover, a set of quality attributes of eggs to which respondents pay attention when they choose eggs were investigated [30,60]. In line with the current literature [59,61], food neophobia and food techno-neophobia were also measured using several items [59] (for details see the Supplementary Materials).

The second part of the survey focused firstly on the willingness of people to eat eggs enriched with omega 3. Consumers had to indicate their familiarity towards functional eggs; the survey, furthermore, asked respondents if they had ever consumed eggs enriched with omega-3 in the past. Moreover, following the previous literature focused on consumers’ behavior [5], consumers’ perceptions of functional eggs were explored using a decimal scale response. The motivation at the base of consumers’ willingness to eat eggs enriched with omega 3 was also analyzed. Furthermore, the willingness of people to pay a premium price for eggs enriched with omega 3 was constructed as the levels of participants’ willingness and was categorized into three different units: none, moderate and high. The group “none” indicated that the respondent was unwilling to pay an additional price for eggs enriched with omega-3, while for the categories “moderate” and “high”, the WTP revealed that respondents were willing to pay a higher price for functional eggs (up to 50% and >50%, respectively) in comparison with the price of the conventional egg, which was considered to be EUR 0.32 [62].

Finally, following the current literature about the topic [4,60], information about socioeconomic aspects of the sample, such as age, gender, education, marital status and annual income were collected (for details about the questionnaire see the Supplementary Materials).

It is important to underline that the questionnaire was validated on 50 respondents before being used in the survey [63,64].

All statistical analyses were performed using R, Version 3.6.2 [65].

### 3.2. The Factor Analysis

A factor analysis with orthogonal rotation (varimax) was applied to reduce food neophobia and food techno-neophobia groups to a smaller set of summary variables and, later, to include them in the econometric model. In particular, Table 1 shows the two factors included in the analysis with their Cronbach’s *a* values. In particular, the first factor is called *neophobia* with a Cronbach’s *a* of 0.96 after having removed five items with factor loadings less than 0.60, while the second factor is named *food techno-neophobia* with a Cronbach’s *a* of 0.90 after having excluded one item with a factor loading less than 0.60.

Finally, the mean sum of the constructs was conducted and used in the econometric model.

### 3.3. The Econometric Model

To identify which factors drive the consumers’ WTP for eggs enriched with omega-3, a Tobit regression model was used [66]. In particular, the Tobit model is applied to understand if a given parameter explains consumers’ willingness to pay variations when the other variables are controlled [67]. The Tobit model was used because the dependent variable showed a substantial share of zero in the data (25% of the sample) and the rest with a positive level of WTPs (Table 2). This choice depends on Tobit model characteristics, which allow us to account for zero-value observations of the dependent variable. In fact, Tobit model is usually applied to estimate equations that have dependent variables that are continuous over some range, while are censored at lower end. In our case, about 75% of consumers claimed positive values for willingness to pay for functional eggs, while almost 25% of the sample was not willing to pay a higher price for them, consisting in a censored dependent variable with a limited value at zero.

For these reasons and following Cameron and Trivedi’s study [66], the regression of interest were considered as an unobserved latent variable y*, as follows:

(1) $$y^{*}=x_{i}^{\prime }\beta +\varepsilon_{i},\;i=1,\ldots .,N$$

where $\varepsilon_{i}\sim \;N\left(0,\sigma^{2}\right)$ and x_i_ is the (K × 1) vector of exogenous and fully observed regressors. The observed variable y_i_ is related to the latent variable $y_{i}^{*}$ through the observation:

(2) $$y=\;f\left(x\right)=\left\{\begin{array}{c} L\;,\;\mathrm{if}\;y^{*}\leq L\\ y^{*},\;\mathrm{if}\;y^{*}>L \end{array}\right.$$

The probability of a variable to be censored is P(y* ≤ L) = P(x’β + ε_i_ ≤ L) = Φ{(L − x’_i_β)/σ}, where Φ(∙) is the standard normal cumulative distribution function. Moreover, the expected value of y for non-censored observations is written as:

(3) $$E\left(y_{i}{|x}_{i},{\;y}_{i}>L\right)={\;x}_{i}^{\prime }\beta +\sigma \frac{\Phi \left\{\left(x_{i}^{\prime }\beta -L\right)/\sigma \right\}}{\Phi \left\{\left(L-x_{i}^{\prime }\beta \right)/\sigma \right\}}$$

where Φ(∙) is the standard normal density, and this last equation relies on the assumption that $\varepsilon_{i}\sim \;N\left(0,\sigma^{2}\right)$.

## 4. Results

Considering the 312 people interviewed, 58% were women, while males accounted for 42% of the sample. The respondents’ age was in the range from 18 to 70 years old, with an average value of about 34 years old, reporting a standard deviation of 11.9. The education level of the respondents was generally high; indeed, 49.7% of participants had a university degree, 11.2% had a master’s degree or PhD and only 39.1% of respondents had attended an upper secondary school as their highest education level. Moreover, the result shows that the majority (66.34%) of egg consumers were not married and about 30% of the participants showed a yearly income ranging between EUR 20,001–30,000, followed by 28.20% of respondents with an annual income in the range EUR 10,001–20,000. Moreover, about 75% of participants were willing to pay for functional eggs enriched with omega-3-fatty acids.

The variables included in the Tobit model (Table 3) approximate the attitudes of respondents and several quality features able to affect participants’ WTP for eggs enriched with omega-3-fatty acids. In more depth, the latent variable WTP for functional eggs increases with the rise in all the explanatory parameters, except for the neophobia and techno-neophobia factors, gender and marital status of the respondents. Among the eggs’ attributes, only color of the shell and color of the yolk were not statistically significant.

According to the findings, the WTP for eggs enriched with omega-3-fatty acids increased with the growing importance that people attributed to the size of eggs, rearing type, feed given to chickens, the provenience (i.e., local, national or international provenience) and brand of eggs. Moreover, it is important to observe that the WTP for functional eggs decreased with the growth in neophobia and food techno-neophobia factors, and this result could be due to the fact that consumers perceived eggs enriched with omega-3-fatty acids as a potential new food. Among the socio-demographic features of the sample, just gender and marital status decreased with the rise in WTP, indicating that unmarried women were more willing to pay a higher price for functional eggs than other consumers. Finally, the positive sign for respondent’s income revealed that the probability of having a higher willingness to pay for functional eggs increased among consumers who had a high level of annual income.

## 5. Discussion

### 5.1. Remarks

The potential demand for functional food is often complicated to assess due to the non-availability of actual market data [68]. Consequently, hypothetical and non-market valuations of new functional foods by consumers are often applied to retrieve useful information [69]. The WTP approach is very important for analyzing the market of differentiated products [70,71]. The current literature suggested that poor attention has been paid to eggs enriched with omega-3-fatty acids. In fact, to our knowledge, no study has paid attention to Italian consumers’ preferences for functional eggs. This gap led authors to question which egg quality characteristics, consumers’ attitudes and socio-demographic characteristics could affect consumers’ WTP for eggs enriched with omega-3-fatty acids. The limited references about purchasing functional eggs support the importance of this study, intending to verify if eggs enriched with omega-3-fatty acids are of interest to Italian consumers.

In the introduction of new foods (such as functional foods), a price assessment needs to be carried out to determine the ability of customers to pay for these products [72]. In general, consumers of functional foods tend to pay a reasonable price to consume them [46,73,74]. In our case, the majority of respondents agreed to pay a reasonable premium price for functional eggs enriched with omega-3-fatty acids. In fact, 69% of respondents were willing to pay a price ≤50% more for a functional egg in comparison to the price of a conventional egg considering a price of EUR 0.32 [62], while 6% of the sample were willing to pay a premium price >50%.

The current literature has demonstrated that the acceptance of functional food by consumers can be influenced by several different factors, and among these, socio-demographic features of people such as gender, marital status and income are important drivers. [4,6,31,35]. The findings showed that unmarried females are more willing to pay a premium price for functional eggs than male consumers, and the probability of having a higher WTP for functional eggs increases among consumers with a high annual income. These results were in line with the current literature (for a review see [4,35]) where, according to some authors [31], women are more willing to pay a premium price for functional foods. Similar results were reached by other authors [2,51,54] who found that females were more likely to eat functional foods than males, and this could be due to both their higher interest in healthy eating and their greater attention in the control of body weight [75]. Furthermore, Bekoglu et al. [48] showed that the marital status of people affected consumers’ acceptance of functional foods. In fact, they [48] showed that single consumers were more likely to consume functional foods than married people. According to other authors [4,31,53,56,57], a higher income level is positively associated with higher purchase intentions of people, as shown in our case. Similar results were reported by Ali and Ali [76], who showed that the income of people is an important demographic parameter, which is more likely to influence the consumers’ WTP for healthy food products as functional food.

In addition, the brand and product characteristics (e.g., size of eggs) are important factors that influence consumers’ functional food choices [44,45,46], and, in particular, these drivers strengthen consumers’ perceptions of the quality of the eggs [72,77]. In our case, about 71% of the respondents stated that they pay attention to the brand of the eggs and about 53% pay attention to the size of the eggs when purchasing them. Similar results were reached by some authors [60], who found that 90% of their sample mentioned paying attention to the brand when purchasing eggs. In our case, the size and brand of the eggs were important variables in the WTP for eggs enriched with omega-3-fatty acids. Similar results were reached by Mirosa et al. [46] who showed that people with knowledge about the brands tended to eat more functional foods. Moreover, in our case, the fact that consumers expressed a specific interest in the size and brand of eggs implies that these aspects were considered as a quality attribute of eggs. Similar results were shown by Relawati et al. [72] who found that the brand strengthens consumers’ perceptions of the quality of the eggs. In fact, the brand could improve customers’ intention, motivation and trust in the authenticity and quality of eggs, which lead to the willingness to pay [72].

Moreover, information regarding the provenience of eggs (i.e., local, national or international provenience of eggs) are important aspects for consumers’ willingness to pay [78]. In fact, according to Gracia et al. [78], consumers are willing to pay a higher price for the geographic distance from the place of production (i.e., local, regional and national over imported). Additionally in our case, the proximity of production (provenience of eggs) is an important variable in the econometric model. In fact, the WTP for eggs enriched with omega-3-fatty acids increases with the growing importance that people attributed to the provenience (i.e., local, national or international provenience) of eggs. According to Gracia et al. [78] locally-produced eggs can be used to differentiate products in the market. In our case, this might mean that if functional eggs were locally produced, a new market segment would be created.

Furthermore, on the one hand, an aversion to new food could lead people to be reluctant to consume some functional foods [4]. On the other hand, the process of producing new functional food applies food technologies that could be unfamiliar to consumers, leading people to be skeptical or reluctant to consume some functional foods [4]. In other words, food neophobia and food techno-neophobia are obstacles for consumers’ acceptance of new foods [79,80]. As expected, in our case, food neophobia and food techno-neophobia decreased the people’s WTP for enhancement of eggs with omega-3. These results might be explained by what was reported in previous studies [10,32,48,51]. In fact, according to some authors [32,51], food neophobia causes a negative effect on consumers’ attitude towards functional foods. Saher et al. [10] showed that people who buy functional products are regarded as more innovative (i.e., explorative people towards new food) than consumers of conventional foods; vice versa Bekoglu et al. [48] found that people who are innovative are more likely to consume functional foods. Additionally, in our case, consumers who are willing to pay a premium price for functional eggs might be regarded as more innovative (i.e., neither neophobic or techno-neophobic people) than consumers of conventional foods. According to Dolgopolova et al. [81], a high amount of food neophobia was related to the fact that consumers perceive conventional food as the most important guarantee for healthy food. On the other hand, food techno-neophobia can also be a critical factor in facilitating the spread of functional food [82]. In fact, consumers’ acceptability of functional food depends on their knowledge about the technology used to produce them [9] and the ingredients used [4]. In particular, according to some authors [4], consumers were more likely to accept functional foods with natural enrichments. In our case, the results revealed a clear necessity by consumers to retrieve information regarding the rearing type (i.e., free-range eggs, barn and caged eggs) and feed given to chickens (variable called *animal_feed*) confirming the current literature about functional food [4,35]. In fact, according to some authors [83], consumers are strongly interested in rearing types (i.e., method used), while other authors [84] showed that consumers have a misunderstanding about rearing type and, thus, the relative benefits on human health of the different types of eggs. Goddard et al. [84] found limited consumer interest in free-range eggs (i.e., rearing types), identifying small niches for this type of egg, while Gracia et al. [78] showed that consumers are willing to pay a higher price for an enhanced method of production (that of barn and/or free-range instead of cage produced eggs). Another important factor that drives the respondents’ willingness to pay a premium price for eggs enriched with omega-3-fatty acids is information about the feed given to chickens (i.e., ingredients used for producing functional eggs). These findings confirms the current literature about consumers’ behavior [4,6,15], which emphasizes the necessity for people to have information about new food production and the ingredients used. The results suggest that consumers need to have clear information about functional eggs, after which, information on the production of functional eggs might be more effective [30]. This showed the importance of providing this type of information to consumers. In fact, consumers make the decision on consumption with regards to the level of information they have in order to maximize their utility [60,85]. According to Mesìas et al. [30], consumers’ lack of knowledge regarding the features of these products, and about how they differ from conventional items, represents one of the major constraints to obtaining a share of the market. In fact, proper knowledge and communication seem to be the most reliable ways to increase people’ interest in functional products [35].

### 5.2. Practical Implications

The study’s results might suggest the attractiveness of functional eggs in Italy. The functional eggs concept might have potential appeal in Italy, and the findings should be useful to draw up marketing strategies for firms. In fact, the study objective is fundamental and a prerequisite to developing successful marketing strategies, which, by default, constitutes the primary value and contribution of this work. The present study should have important practical implications for firms and industries in the food sector interested in the production of functional food. The findings show the substantial potential for eggs enriched with omega-3-fatty acids, which seems to be a product that has a good chance to be satisfactorily appreciated on the market, especially if accompanied by an informative campaign in order to increase consumers’ knowledge level.

### 5.3. Limits and Future Research

This study was conducted to understand if eggs enriched with omega-3-fatty acids were of interest to Italian consumers, and to analyze which characteristics of table egg quality, consumers’ attitudes and socio-demographic characteristics affect the consumers’ willingness to pay (WTP) a premium price for functional eggs. However, a limitation of our study was that the limited market share and lower awareness level of functional foods by consumers caused lower attendance for this survey [47]. Moreover, we are aware that the conclusions of this study cannot be overgeneralized since the interviewees were engaged through a web-based survey on a voluntary basis. Thus, a further study based on a representative population is needed to ensure the overall validity of the findings. Further research should also be focused on firms in the food sector interested in functional foods to understand if the individuated price in the current study might be profitable for firms, in order to offer the opportunity to retrieve some new insights and to propose further discussion on a functional food such as eggs enriched with omega-3-fatty acids.

## 6. Conclusions

New trends in the market, as well as the increasing attention for functional foods, are opening up new possibilities for food products. This especially applies to eggs, which are a staple part of the Mediterranean diet, in particular in Italy the where Mediterranean diet is strongly rooted.

Even if the interest for functional food has strongly increased, some factors, such as the lack of a clear definition, a dedicated regulation and their technological description, are jeopardizing their effective development. The different stakeholders, in fact, speak different languages, creating difficulty for functional food development. For these reasons, a holistic approach to functional food implementation is required. In fact, first, regulation and definitions should be taken into consideration; technological aspects of functional foods should be described. Finally, functional foods on the market require dedicated communication strategies. The study’s findings showed the importance of communication for consumers; in fact, eggs enriched with omega-3-fatty acids seems to be a product with good possibilities to be appreciated on the market, especially if an efficient, informative strategy is implemented. This aspect is important because consumers make their decision on consumption with regards to the level of information they have; on the other hand, firms belonging to the food sector require information about consumers’ needs, thus representing an important input for involved stakeholders to drive the development of functional food, setting it within an iterative and virtuous holistic cycle.

## Figures and Tables

**Table 1:** The factor analysis for food neophobia and food technology neophobia groups.

Items Group	Neophobia	Technology Neophobia
Food neophobia (σ = 0.96)		
I am constantly sampling new and different foods (new_food *)	0.86	
I like foods from different cultures (different_culture *)	0.93	
At dinner parties, I will try a new food (dinner_try *)	0.87	
I like to try new ethnic restaurants (restaurant *)	0.90	
Food technology neophobia (σ = 0.90)		
New food technologies are unnecessary (no_new_tec)		0.76
The environmental benefits of new food technologies are often overstated (env_tec)		0.92
The benefits of new food technologies to reduce world hunger are often overstated (hunger_tec)		0.91
New food technologies decrease the natural quality of food (low_quality)		0.84
There is no sense in trying out high-tech food products because the ones I eat are already good enough” (good)		0.73

* Reversed coded. Source: Our elaboration on the survey data.

**Table 2:** The WTP for functional egg in comparison to the price of conventional one considering a price of EUR 0.32 [62].

Levels of WTP for Functional Eggs	%
WTP = 0	25.00%
WTP ≤ 50%	69.00%
WTP > 50%	6.00%
Total	100.00

Source: Our elaboration on the survey data.

**Table 3:** Results of the Tobit regression model.

Tobit Regression		N. obs=	312
Log-likelihood: −274.832		Pseudo R^2^=	0.15
Dept. Variable: WTP functional eggs			
**Variables**	**Coeff.**	**Std. Err**	***p*_value**
Intercept 1	−0.321	0.203	1.15 × 10^−1^
Intercept 2	−0.562	0.044	2.00 × 10^−16^ ***
eggs size	0.099	0.034	3.00 × 10^−3^ **
color shell	0.085	0.044	6.30 × 10^−2^
rearing type	0.089	0.026	6.00 × 10^−4^ ***
provenience	0.197	0.057	6.00 × 10^−4^ ***
color_yolk	−0.014	0.021	4.91 × 10^−1^
animal_feed	0.077	0.019	5.81 × 10^−5^ ***
brand_prod	0.067	0.021	1.90 × 10^−3^ **
neophobia	−0.078	0.023	8.00 × 10^−4^ ***
food techno-neophobia	−0.037	0.015	1.60 × 10^−2^ *
gender	−0.142	0.072	4.90 × 10^−2^ *
marital status	−0.253	0.071	4.34 × 10^−4^ ***
income	0.083	0.029	4.37 × 10^−3^ **

Note: ***, **, * Significance at 0.001, 0.01 and 0.05 levels. Source: Our elaboration on the survey data.

## Data Availability

Data is contained within the article.

## Supplementary Materials

The following are available online at https://www.mdpi.com/article/10.3390/foods11040545/s1, Table S1: Variables used in the questionnaire.
