# Supplementary material for: Factors Influencing Italian Consumers’ Willingness to Pay for Eggs Enriched with Omega-3-Fatty Acids
Source: Foods. 2022 Feb 14;11(4):545. doi: 10.3390/foods11040545 (PMC8870929; doi:10.3390/foods11040545)
Supplement: Supplementary file 1 [file foods-11-00545-s001.zip › foods-1581213-supplementary.pdf]

Supplementary Materials

Factors Influencing Italian Consumers' Willingness to Pay for Eggs Enriched with Omega-3-Fatty Acids

The questionnaire

Table S1. Variables used in the questionnaire.

Item Groups	Items	Issues
		**Section 1: consumers habits, their preferences and their attitudes towards food choice (mean score on 10-point scale)**
1		You are: omnivore (ab) (0); vegetarian (1); vegan (2)
2		Responsibility for the grocery shopping in family (buyer) (0 = no, 1 = yes)
3	Frequency of some food consumption (0 = never; 1 = once a month; 2 = once a week; 3 = twice a week; 4 = more than twice a week; 5 = every day)	Red meat (red_meat)
		White meat (white_meat)
		Dairy products (dairy)
		Eggs (eggs)
		Legumes (legumes)
		Fish (fish)
		Dry fruit (fruit)
		Vegetables (vegetables)
		Fresh fruit (fresh_fruit)
		Cereals (cereals)
4		Do you do gym? (fisic) (0 = No; 1 = Yes)
5		Do you suffer from cardiovascular disease? (cardio) (0 = No; 1 = Yes)
6		Do you suffer from hypercholesterolemia? (ipercol) (0 = No; 1 = Yes)
7		Do you suffer from blood pressure? (press) (0 = No; 1 = Yes)
8	Aspects you pay attention when you consume food (Health aspects)	The hygienic requirements of food (hygienic)
		The effects on human health (health_effects)
		The caloric intake of food (caloric)
		The food protein intake (proteic)
		The food fat intake (fat)
		The environmental impact of food consumption (environmental_impact)
		The geographic origin of food (geographic)
		The food and culinary traditions (traditions)
		The production method of food (production_method)
9	How the environmental issues influence your food choices? (Env_aspects)	I inform on low impact products (low_impact)
		I inform on provenience of products (or)
		I know brands with low environmental impacts production (brand_low_impacts)
		I always buy organic products (buy_organic)
		I always read label products with attention (label)
		I try to buy products on local market (local_products)
		I try to buy seasonal products (season_prod)
		My food choices are not influenced by the environmental issues (no_inflence)
		I avoid brands of foreign products (foreign_prod)
		I try to reduce meat consumption (reduce_meat)
10		Where do you buy eggs? (where) (1 = by a breeder; 2 = in a supermarket; 3 = I'm a breeder; 4 = other)
11	Attributes you pay attention when you choose table eggs (Attributes of eggs)	Eggs size (eggs size)
		Color shell (color shell)
		Rearing type (i.e. free-range eggs, barn and caged eggs) (rearing type)
		Color yolk (color_yolk)
		Feed given to chickens (animal_feed)
		Brand (brand_prod)
		Provenience of eggs (i.e. local, national or international provenience of eggs) (provenience)
12	Agreement or disagreement with the following statements (Food neophobia)	I am constantly sampling new and different foods (new_food*)
		I don't trust new food (dont_trust)
		If I don't know what is in a food, I won't try it" (no_try)
		I like foods from different cultures (different_culture*)
		Ethnic food looks too weird to eat (strange)
		At dinner parties, I will try a new food (dinner_try*)
		I am afraid to eat things I have never had before (fear)
		I am very particular about the foods I will eat (attention)
		I like to try new ethnic restaurants (restaurant*)
13	Agreement or disagreement with	New food technologies are unnecessary (no_new_tec)
		The environmental benefits of new food technologies are often overstated (env_tec)

	the following statements (Food technology neophobia)	The benefits of new food technologies to reduce world hunger are often overstated (hunger_tec)
		New food technologies decrease the natural quality of food (low_quality)
		There is no sense in trying out high-tech food products because the ones I eat are already good enough" (good)
		I don't have enough knowledge on effects of new food technologies on human health (effect_health)
**Section 2: consumer perceptions and their attitudes towards functional eggs**		
14		Have you ever heard about functional food? (func_food) (0 = No; 1 = Yes)
15		Have you ever heard about functional eggs enriched with omega 3? (egg_omega) (0 = No; 1 = Yes)
16		Do you know production method of eggs enriched with omega 3? (method_egg) (0 = No; 1 = Yes)
17		Have you ever eaten functional eggs enriched with omega 3 before? (pass) (0 = No; 1 = Yes)
18		Would you be willing to eat functional eggs enriched with omega 3? (will) (0 = No; 1 = Yes)
19		How much would you be willing to pay for functional egg enriched with omega 3 compared with that one conventional egg (whom price is of 0.32 euro)? (wtp) (0 = none, 1 = ≤50%, 2 = >50%)
20	What functional egg characteristics could affect your decision to eat it? (Functional eggs)	If I think to eat functional egg enriched with omega 3, I'm curious to try it (curiosity)
		If functional egg enriched with omega 3 produces health benefits, I would eat it (low_col)
		If my friends eat functional egg enriched with omega 3, I would eat it (friends)
		If functional eggs enriched with omega 3 are more nutrient than conventional eggs, I would eat it (more_nutrient)
		If functional egg enriched with omega 3 is less expensive than conventional egg, I would eat it (low_cost)
		If functional egg is produced by my trusted farmer, I would eat it (farmer)
		If I get more information about functional eggs enriched with omega 3, I would eat it (more_info)
**Section 3: sociodemographic information**		
21		Age (age)
22		Gender (gender) (0 = female; 1 = male)
23		Education (edu) : 0 = primary or secondary (low education); 1 = degree, master and/or PhD (high education)
24		Marital status (marital status) (0 = unmarried; 1 = married; 2 = separated/divorced)
25		Annual Income (income) (0 = <10,000; 1 = 10,001–20,000; 2 = 20,001–30,000; 3 = 30,001–40,000; 4 = 40,001–50,000; 5 = >50,001)

*Reversed coded.

Word in brackets refers to the abbreviation of the variable in the manuscript.
